# Socio-demographic and environmental determinants of under-five child mortality in Ethiopia: using Ethiopian demographic and Health 2019 survey

**DOI:** 10.1186/s12887-023-04026-w

**Published:** 2023-06-15

**Authors:** Meseret Girma, Hiwot Eshete, Rahel Asrat, Mignote Gebremichael, Dereje Getahun, Tadesse Awoke

**Affiliations:** 1 Institute of Public Health, Collage of Medicine and Health Sciences, University of Gonder, Gonder, Ethiopia; 2 School of Nutrition, Food science and Technology Hawassa University, Hawassa, Ethiopia

**Keywords:** Child mortality, Ethiopia

## Abstract

**Background:**

The under-five mortality rate is a reliable indicator of a country’s general level of development and the wellbeing of its children. Life expectancy is a good indicator of a population’s standard of living.

**Objectives:**

To identify Socio-demographic and Environmental determinants of under-five child mortality in Ethiopia.

**Methods:**

A national representative cross sectional study and a quantitative study were conducted among 5753 households selected based on 2019 Mini-Ethiopian Demographic and Health Survey (EDHS-2019) data. The analysis was done using STATA version 14 statistical software. Bivariate and multivariate analyses were used. To assess the determinants of under-five child mortality in multivariate analysis, p values less than 0.05 were considered statistically significant, and odds ratios with 95% CI (confidence interval) were used.

**Results:**

A total of 5,753 children were included in the study. Sex of the head of the household being female (AOR = 2:350, 95% CI: 1.310, 4.215), the U5CM for being their mother were currently married (AOR = 2:094, 95% CI: 1.076, 4.072), The odds of U5CM was less by 80% (AOR = 1.797, 95% CI: 1.159–2.782) with the number of under-five children at the household born 2-4th order as compared to those children born on the first order. For the mothers visit anti natal care four and above visit (AOR = 1:803, 95% CI: 1.032, 3.149), for the way of delivery (AOR = 0:478, 95% CI: 0.233, 0.982).

**Conclusion:**

Multivariate logistic analysis reflected that way of delivery, mothers had being currently marred, sex of the head of the household and number of antenatal care visit were found to be significant predictors of under-five child mortality. So, government policy, nongovernmental organizations, and all concerned bodies should be focused on the major determinants of under-five child mortality and put in a lot more effort to reduce under-five child mortality.

## Introduction

Between 1990 and 2015, the Millennium Development Goals (MDGs) set a goal of halving child mortality by two-thirds, with a reduction in under-five mortality rate as one of the indicators [1].

Since 1990, the mortality rate of children under the age of five has decreased by 35% in developing countries, from 97 deaths per 1000 births to 63. Between 1990 and 2000, the average rate of reduction in child mortality in Sub-Saharan Africa was 1.2% per year, and between 2000 and 2010, it was 2.4%. Children in impoverished countries are twice as likely as children in developed countries to die before reaching their fifth birthday [2].

“The enjoyment of the highest achievable standard of health is one of the fundamental rights of every human person, regardless of race, religion, political belief, economic or social position,“ according to the World Health Organization (WHO). To achieve this goal, the WHO encourages people to work on social determinants of health in order to reduce inequity and injustice caused by people’s socioeconomic background [3]. Under-five children are not also immune to these social determinants due to their age (voiceless to make decisions) and their parents’ social background.

The interplay of several factors causes under nutrition in children, including maternal age, maternal education, poor feeding practice, maternal nutritional status, parity and multiple births, sex of the child, illness, birth interval and immunization status, poor wealth status, large families, water and sanitation, place of residence, and other factors relating to health service utilization [4–7].

Child malnutrition and mortality rates are reasonable responses to both well-being initiatives and economic factors including pay, unemployment, and lack of education [7].

Higher national incomes were related with reduced under-5 mortality rates, according to a cross-national comparative analysis of the determinants of under-5 mortality among 43 developing nations [8].

Despite the fact that childhood mortality is rapidly declining and access to and utilization of health care is improving in Ethiopia, but the study found a high prevalence of under-five mortality, 8.7% (87/1000) in southeastern Ethiopia [9].

Despite a significant decrease in under-five mortality in Ethiopia, a significant number of under-five children were still dying. Early breastfeeding initiation, the preceding birth interval, and adolescent pregnancy were all preventable causes of under-five mortality [10].

Higher birth order, no maternal education, and lower wealth quintile were all linked to a higher risk of under-five mortality in a study based on data from the 2003 Nigerian Demography and Health Survey (DHS) [11]. Also, in Bangladesh and Sudan, a similar study discovered nearly identical determinants of under-five mortality [12, 13]. The factors of under-five mortality differ in rural and urban settings, according to data from the 2008–2009 Kenya DHS and Zhejiang Province in China [14, 15].

Children in rural areas are more likely to die, according to the United Nations (UN) 2012 report on the Millennium Development Goals (MDGs). Children born into poverty are almost twice as likely as those from wealthier families to die before reaching the age of five. Access to schooling by mothers is a survival factor for children under the age of five [2].

Under-5 mortality declined from 166 deaths per 1,000 live births in 2000 to 67 deaths per 1,000 live births in 2016 and 48.7 deaths per thousand live births in 2020. This represents a 60% decrease in under-5 mortality over 16 years. Moreover, the mortality rates distribution differs by geographical regions.

The determinants of child and under-5 mortalities are highly correlated with socioeconomic, demographic, and behavioural factors of households, health seeking behaviour of mothers, and environmental factors. Several studies have found that maternal age and education are strongly related to child morality [16]. Level of education is inversely associated with child mortality children of educated mothers having better survival than children of non-educated mothers [17].

According to the United Nations Children’s Fund (UNICEF), has re-ported that the number of under-five fatalities worldwide has decreased from roughly 12 million in 1990 to an estimated 6.9 million in 2011 [18]. Ethiopia has made significant progress in reducing infant mortality since 2000, according to new commentary research. The global alliance provided financial and technical support for this achievement. However, by the end of 2019/2020, the SDG targets for under-five mortality reduction have not been met, which could be owing to partnership dynamics caused by the COVID-19 pandemic, or the pandemic itself could damage the SDG target [19].

Access to health-care services increased the likelihood of a child’s survival. Children of mothers who did not receive prenatal and postnatal care had a higher risk of dying before the age of five than children of mothers who did receive care. Most studies also found that sex of the household head, sex of the child [17, 20], and household economic status (wealth index) were found to be significant [20], where male children are at higher risk of under-five mortalities.

Understanding the social determinants of under-five mortality in Ethiopia can help determine which parts of the population need support in order to reduce mortality rates more quickly. Despite the fact that a number of studies have been conducted to identify factors associated with under-five mortality in Ethiopia, progress in decreasing child mortality remains high, and more effort is required to remove barriers to under-five survival. More research is needed to inform policymakers on how to implement appropriate health intervention programs in order to meet the Sustainable Development Goals (SDGs), which aim to reduce under-5 mortality rates to less than 25 deaths per 1,000 live births by 2030. To address this gap, we conducted an all-inclusive cross-sectional analysis of the most recent 2019 Ethiopian Demographic Health Survey, to investigate the major risk factors for under-five mortalities in Ethiopia, taking into consideration various demographic, socioeconomic, and environmental factors.

Therefore, the main objective of this study is to assess the socio demographic and environmental determinant of under-five mortality in Ethiopia based on Ethiopian Demographic Health Survey, 2019 dataset.

## Methods and materials

### Study setting and period

Secondary data analysis was conducted based on the EDHS 2019, which is the fourth survey conducted nationally from March 21 to June 28, 2019.

It has nine regional states (Tigray, Afar, Amhara, Oromiya, Somalia, Benishangul-Gumuz, Southern Nations Nationalities and People [SNNP], Gambela, and Harari) and two city administrations (Addis Ababa and Dire Dawa).These are further subdivided into 68 zones, 817 administrative districts, and 16 253 Kebeles, the country’s smallest administrative units.

Ethiopia had an estimated population of 114.96 million in 2020, making it the second-most populous country in Africa after Nigeria and the world’s 12th-most populous country.

This study used a publicly available dataset from the 2019 Ethiopian Demographic and Health Survey (EDHS). website https://www.dhsprogram.com/data/dataset/Ethiopia_Interim-DHS_2019.cfm?flag=1.

### Study design

A Community-based Cross-sectional data came from Ethiopia’s Mini Demographic and Health Survey 2019. The Ethiopian Public Health Institute (EPHI) worked with the Central Statistical Agency (CSA) and the Federal Ministry of Health (FMoH) to conduct the 2019 Ethiopia Mini Demographic and Health Survey (2019 EMDHS), which was coordinated by the Technical Working Group (TWG) from March 21 to June 28, 2019.

### Study population

All live births in the five years preceding the survey period in the selected Enumeration Areas were included in the study populations (EAs). The required sample size was extracted from the children’s record (KR) file from the standard DHS dataset. Finally, a total of 5753 under-five children were included in this study.

### Study variables

#### Dependent variable

In this study, the dependent variable was under five children aged < 5 years, which is dichotomized in to “Yes” if the child is alive and “No” if the child is died.

#### Independent variables

Age of child, Sex of child, Marital status, religion, Child spacing, sex of household head, Educational status of the mother, Number of under-five child in a house, place of delivery, delivery by cesarean section, current contraceptive status, current marital status, number of ANC visits, birth order number Source of drinking water, Availability of toilet facility.

### Sample size sampling technique

A two-stage cluster sampling technique was used to collect and stratify the EDHS samples. After stratifying each region into urban and rural, 305 (93 urban 212 rural) clusters or enumeration areas were chosen in the first stage.

A fixed number of 30 households per cluster were chosen with an equal probability of systematic selection from the newly created household listing in the second stage of selection. All women between the ages of 15 and 49 who were either permanent residents or visitors who slept in the selected households the night before the survey were eligible to be interviewed. All children aged 5 years old within 5 years of the surveys in Ethiopia were the population source for this study, whereas all children aged 5 years old in the selected enumeration areas within 5 years of the survey were the study population. The 2019 survey included a total representative sample of 5753 children aged 5 years.

The sample included 5,753 EAs, 1,328 in urban areas and 4,425 in rural areas.

### Operational definitions

#### Under-five mortality

the probability of a child dying between birth and exactly 5 years of age, expressed per 1,000 live births.

### Data collection tool and procedures

Secondary data was used for this study that obtained from 2019 EDHS. The surveys were conducted by the Central Statistics Authority (CSA) Federal Democratic Republic of Ethiopia. The data set consists of a national representative sample of household level data.

### Data processing and analysis

After requesting and receiving permission from the Mini-Demographic and Health Survey (EDHS) Program, the EDHS-2019 dataset was downloaded electronically by logging at https://dhsprogram.com/Data/terms-of-use.cfm. Data management and analysis was carried out using STATA 14 software. Descriptive statistics were done to assess basic client characteristics. Bivariate analysis using logistic regression technique was done at P-value less than 0.25 to select the candidate variables to be entered into multiple logistic regression models for controlling confounding factors.

The model was evaluated using the backward stepwise selection method and the model fitness was assessed using the Hosmer and Lemeshow goodness-of-fit test. Interpretations of the strength of the associations between associated risk factors and the response variable were based on significant adjusted odds ratios (AOR) with their respective 95% confidence intervals at 5% level of significance (*p*-value < 0.05).

#### Ethical consideration

After receiving ethical authorization from the International Review Board of Demographic and Health Surveys (DHS) program data archivists of written informed consent for this project, the survey was conducted. The dataset is publicly available when looking for a concept note for a proposed project.

## Results and discussions

### Result

The initial population consisting of 5, 753 children’s information was obtained by interviewing face to face their mothers and have complete measurements and were considered in this study.

Higher U5CM were observed in a family currently not married 31(7.79) than the family currently married 308 (5.75%).

Lower under-five mortality occurred in Orthodox (71 (4.40%)) and Protestant (50 (4.75%)) compared with Muslim (210 (7.06%)) and higher death observed among traditional religion followers.

Of the total of 5,753 children included in the study, 1,328 (23.08%) were born in urban and 4,425 (79.92%) were born in the rural part of Ethiopia. The higher percentage of death rate (267 (6.03%) among children under five was recorded in the rural area, when compared to urban areas (72 5.42%).

The use of contraceptive method had shown to less the child death 85(4.52%) than the mother who were not use any contraceptive 254 (6.56%).

From the total deaths, 86 (6.82%) occurred with the first birth order, 119 (4.58%) death occurred between the second and fourth birth order and the rest 134 (7.07%) deaths were found from the birth orders five and above. The child mortality rate is thus confirmed to be increasing steadily with birth order. The increase in the child mortality rate with birth order may reflect a more intense competition faced by higher birth order of children in terms of caregiver time, medical resources, and nutritious food which are required by children.

The higher U5CM were observed among mother’s had history of “Don’t know anti natal care” 2(11.76%) followed by with mothers had no ANC visit 79(7.57%) and the under-five mortality were shown to decreased as the ANC follow up 1 visit 6 (4.26), 2–3 visit 38(3.39%), and + 4 visit 54 (3.26%).

From the total number analyzed, 5,753 (50.07%) children were born at home, 2,563 (44.55%) children were born at a public facility, and the remaining 309 (5.37%) children were born at private and other health facilities (Table 1).

197 (6.84%) children died from home delivery, 128 (4.99%) of death occurred from birth in public sectors, and 14 (4.53%) of child death occurred from birth in private sectors.

148 (5.74%) children died to mothers who had a piped source of drinking water, 56 (5.98%) with a spring source of drinking water, and 55(6.33%) who used tube well water.

Child death occurred in those 148 (5.75%) had no toilet and 175 (5.94%) deaths occurred in those with pit toilet facility (Table 1).

**Table 1:** The distribution of child mortality by socioeconomic, bio demographic, environmental, and maternal health care variables in Ethiopia, 2019

Covariates (explanatory variables)	Categories	Under-five child mortality		
		Live	**Death(% of U5CM)**	Total
Sex of household head				
	Male	4,319	**279(6.07%)**	4,598
	Female	1,095	**60(5.19%)**	1,155
Mother’s educational level				
	No education	2,958	**191(6.07%)**	3,149
	Primary	1,701	**122(6.69%)**	1,823
	Secondary	463	**17(3.54%)**	480
	Higher	292	**9(2.99%)**	301
Current marital status				
	Currently not married	367	**31(7.79%)**	398
	Currently married	5,047	**308(5.75%)**	5,355
Religion				
	Orthodox	1,541	**71(4.40%)**	1,612
	Catholic	30	**2(6.25%)**	32
	Protestant	1,003	**50(4.75%)**	1,053
	Muslim	2,764	**210(7.06%)**	2,974
	Traditional	56	**6(9.68%)**	62
	Other	20	**0(0%)**	
Place of residence				
	Urban	1,256	**72(5.42%)**	1,328
	Rural	4,158	**267(6.03%)**	4,425
Current contraceptive method				
	No	3,620	**254(6.56%)**	3,874
	Yes	1,794	**85(4.52%)**	1,879
Birth order number				
	1	1,175	**86(6.82%)**	1,261
	2–4	2,49	**119(4.58%)**	2,598
	5+	1,760	**134(7.07%)**	1,894
Number of ANC visit				
	No visit	965	**79(7.57%)**	1,044
	1 visit	135	**6(4.26%)**	141
	2–3 visit	1,083	**38(3.39%)**	1,121
	4 + visit	1,602	**54(3.26%)**	1,656
	Don’t know	15	**2(11.76%)**	17
Place of delivery				
	Home	2,684	**197(6.84%)**	2,881
	Public	2,435	**128(4.99%)**	2,563
	Private and other facility	295	**14(4.53%)**	309
Delivery by caesarean section				
	No	5,091	**313(5.79%)**	5,404
	Yes	323	**26(7.45%)**	349
Source of drinking water				
	Piped water	2,429	**148(5.74%)**	2,577
	Well water	814	**55(6.33%)**	869
	Spring water	880	**56(5.98%)**	936
	Other	1,291	**80(5.84%)**	1,371
Availability of toilet facility				
	No toilet	2,225	**146(6.16%)**	2,371
	Flush toilet	303	**16(5.02%)**	319
	Pit toilet	2,772	**175(5.94%)**	2,947
	composting	41	**0(0%)**	41
	Other	73	**2(2.67%)**	75

### Factors Associated with Determinants of fewer than 5 child mortality

In bivariate analysis, sex of household head, mother’s educational level, current marital status, religion, place of residence, current contraceptive methods, birth order, number of ANC visit, place of delivery, Delivery by caesarean section, source of drinking water and type of toilet facility had p value less than 0.25. However, in multivariate analysis, sex of household head, current marital status, current contraceptive method, birth order number, number of ANC visit, place of delivery and Delivery by caesarean section had a statistically significant association with U5CM at p value less than 0.05. (Table 2).

The odds of under-five child mortality were 64% (AOR = 1.642, 95% CI: 1.570–2.550) higher among sex of household head being female as compared to being male.

Under-five child mortality currently has married mothers were **2.38** times lower (AOR = 2.387, 95% CI: 1.453–3.921) than among as compared to have not married.

The odds of under-five child mortality were less by 63% (AOR = 1.634, 95% CI: 1.101–2.424) among current contraceptive user mother as compare to non-users.

The odds of under-five child mortality were 80% higher (AOR = 1.797, 95%CI: 1.159–2.782) among children born at 2-4th order as compared to those children born on the first order.

The odds of under-five child mortality were two times lower (AOR = 2.085, 95%CI: 1.342–3.239) among mothers had no ANC visit as compared with those had ANC visit above 2 times.

Children born at private and other facility under-five child mortality were 3.4 times lower than when compared to child born at home (AOR = 3.401, 95% CI: 1.010-11.894).

The odds of under-five child mortality was less by 64% (AOR = 0.353,95%CI: 0.202–0.617) among children delivered by caesarean section as compared with children delivered by non-caesarean section.

**Table 2:** Bivariate and multivariate analyses of variables associated with U5CM in Ethiopia based on EDHS, 2019

Independent variable	Number of children		COR (95% CI)	AOR (95% CI)
	**Alive**	**Death**		
Sex of household head				
Male	4,319	279	1.00	1.00
Female	1,095	60	1.179(0.885–1.570)	1.642(1.57–2.550)**
Mother’s educational level				
No education	2,958	191	1.00	1.00
Primary	1,701	122	0.900(0.712–1.139)	0.695(0.476–1.015)
Secondary	463	17	1.759(1.060–2.916)	1.0309 (0.598-2 0.866)
Higher	292	9	2.094(1.062–4.132)	2.016 (0.583–6.972)
Current marital status				
Currently not married	367	31	1.00	1.00
Currently married	5,047	308	1.384(0.942–2.032)*	2.387(1.453–3.921)**
Religion				
Orthodox	1,541	71	1.00	1.00
Catholic	30	2	0.691(0.162–2.950)	0.979(0.123–17.904)
Protestant	1,003	50	0.924(0.638–1.339)	1.204(0.726–1.997)
Muslim	2,764	210	0.606(0.460–0.799)*	0.789(0.537–1.160)
Traditional	56	6	0.430(0.179–1.034)*	0.675(0.151–3.021)
Other	20	0	1	
Place of residence				
Urban	1,256	72	1.00	1.00
Rural	4,158	267	0.892(0.6839 − 1.167)	1.19(0.769–1.840)
Current contraceptive method				
No	3,620	254	1.00	1.00
Yes	1,794	85	1.491(1.151–1.905)*	1.643(1.101–2.424)**
Birth order number				
1	1,175	86	1.00	1.00
2–4	2,49	119	1.524(1.145–2.029)*	1.797(1.159–2.782)**
5+	1,760	134	0.961(0.726–1.273)	0.165(0.087–0.313)
Number of ANC visit				
No visit	965	79	1.00	1.00
1 visit	135	6	1.842(0.787–4.306)*	1.676(0.704–3.990)
2–3 visit	1,083	38	2.333(1.569–3.469)*	2.085(1.342–3.239)**
4 + visit	1,602	54	2.429(1.703–3.464)*	1.937(1.235–3.149)**
Don’t know	15	2	0.614(0.138–2.733)	0.5156(0.109–2.435)
Place of delivery				
Home	2,684	197	1.00	1.00
Public	2,435	128	1.396(1.110–1.756)*	1.278(0.836–1.951)
Private and other facility	295	14	1.546(0.886–2.695)*	3.401(1.010-11.894)**
Delivery by caesarean section				
No	5,091	313	1.00	1.00
Yes	323	26	0.763(0.504–1.157)	0.353(0.202–0.617)**
Source of drinking water				
Piped water	2,429	148	1.00	1.00
Well water	814	55	0.902(0.655–1.241)	1.020(0.637–1.633)
Spring water	880	56	0.957(0.697–1.315)	1.0081(0.684–1.710)
Other	1,291	80	0.983(0.743–1.301)	1.368(0.876–2.135)
Availability of toilet facility				
No toilet	2,225	146	1.00	1.00
Flush toilet	303	16	1.242(0.731–2.110)	1.230(0.545–2.765)
Pit toilet	2,772	175	1.039(0.828–1.304)	0.901(0.631–1.601)
Composting	41	0	1	
Other	73	2	2.395(0.582–9.856)*	2.062(0.270-15.724)

## Discussions

This study was identifying the determinants of under-five mortality using data from the 2019 EDHS. To investigate factors influencing under-five mortality, descriptive and binary logistic regression analyses were used.

The factors that significantly affect under-five mortality in Ethiopia are sex of household head, current contraceptive method, current marital status, birth order number, number of ANC visit and place of delivery and way of delivery. The odds of under-five child mortality were 64% (AOR = 1.642, 95% CI: 1.570–2.550) higher among sex of household head being female as compared to being male. The female household head was associated with an increase in the incidence of under-five mortality. This finding was consistent with a study done in Ethiopia, Uganda [21, 22]. This finding could be female-headed households are more likely to face food insecurity. And also this indicates that female-headed households will have difficulty making decisions on child health because they will be too preoccupied with other responsibilities in the home and social cases.

Under-five child mortality currently has married mothers were **2.38** times lower (AOR = 2.387, 95% CI: 1.453–3.921) than among as compared to have not married. According to this finding, unmarried women had a higher risk of under-five mortality than married women. This finding is consistent with study conducted in sub-Saharan Africa countries [23]. However, this finding contradicts the findings of other studies [24].

The odds of under-five child mortality was less by 63% (AOR = 1.634, 95% CI: 1.101–2.424) among current contraceptive user mother as compare to non-users. This finding implies that contraceptive use and intention are associated with lower under-five mortality in Ethiopia, and similar findings have been found in Bangladesh and Nigeria. (22, 23). Children of mothers who did not intend to use contraception had a higher risk of dying before the age of five than those who were users or planned to use later. This implies that contraceptive use and met need for family planning have a considerable impact on child survival in Ethiopia. And also may be due to the substantial biological and socioeconomic benefits that are concomitant with contraceptive use which may promote both maternal and child health.

The odds of under-five child mortality were 80% higher( AOR = 1.797,95%CI: 1.159–2.782) among children born at 2-4th order as compared to those children born on the first order. This result was consistent with the results from EDHS 2016 study showed that children with birth order two up to four have a higher risk of dying than a child whose birth order is five and above. And also other study conducted in sub Saharan Africa revealed that the high birth order had a favorable child death than first birth order.

The odds of under-five child mortality were two times lower (AOR = 2.085,95%CI:1.342–3.239) among mothers had no ANC visit as compared with those had ANC visit above 2 times. The study shows that children born from the mother had no ANC follow up were at higher risk of mortality than children born from mothers with had ANC and this is consistently in line with the previous study of 2016 EDHS.

Children born at private and other facility under-five child mortality were 3.4 times lower than when compared to child born at home (AOR = 3.401, 95% CI: 1.010-11.894). This result consistent with the finding of the previous study [25]. This might be due to the fact that children born at home are more susceptible to infections.

The odds of under-five child mortality were less by 64% (AOR = 0.353, 95%CI: 0.202–0.617) among children delivered by caesarean section as compared with children delivered by non-caesarean section. This finding is not consistent with study conducted in Sao Paulo, Brazil [26].

Additionally, a cross-sectional study carried out in India in 2012 found no connection between caesarean deliveries and under-5 mortality [27]. Positive perceptions, such as misunderstanding, fear, and aversion to caesarean section, among mothers may be the possible explanation for the high risk associated with caesarean section in our current study [28].

This study was critical in determining the number of under-five deaths and the factors influencing under-five mortality in Ethiopia. This could be used as a baseline for future prospective studies by researchers. Furthermore, the research will be useful to policymakers, decision-makers, planners, and implementers working in the field.

There are some restrictions and advantages to this study. The estimates may be more generalizable because the study was based on a large, nationally representative dataset. Additionally, this study provides the unbiased true effect size for the determinant factors, to control cluster level and region level dependencies. Data were nevertheless gathered cross-sectionally, which would be subject to bias due to social desirability and recall. The disadvantage of secondary data was unavoidable.

## Conclusions and recommendation

Multivariate logistic analysis reflects that Current marital status, Number of ANC visit, way of delivery, place of delivery, birth order number, contraceptive use, and sex of the head of the household were found to be significant predictors of under-five child mortality. In order to reduce under-five child mortality, government policy, nongovernmental organizations, and all other relevant bodies should concentrate on the key causes of under-five child mortality.

## Data Availability

This study used a publicly available dataset from the 2019 Ethiopian Demographic and Health Survey (EDHS). The sampling and data collection procedure is described in detail on the DHS website https://www.dhsprogram.com/data/dataset/Ethiopia_Interim-DHS_2019.cfm?flag=1”.

## Abbreviations

AOR: Adjusted odds ratio
CI: Confidence interval
COR: Crude odds ratio
CSA: Central statistics agency
EDHS: Ethiopian demographic health survey
EAs: Enumeration areas
MDGs: Millennium development goals
OR: Odds ratio
U5CMR: Under five child mortality rate
UN: United nations
UNICEF: United nations children’s fund
WHO: World health organization
SDGs: Sustainable development goals

## References

[1] Group UND, Fund UNP, Division UNS. Indicators for monitoring the millennium development goals: definitions, rationale, concepts and sources. United Nations Publications; 2003.

[2] Ki-Moon B (2013). The millennium development goals report 2013. United Nations Pubns.

[3] Organization WH (2011). Rio political declaration on social determinants of health.

[4] Black RE, Allen LH, Bhutta ZA, Caulfield LE, De Onis M, Ezzati M (2008). Maternal and child undernutrition: global and regional exposures and health consequences. The lancet.

[5] Mullero K. Malnutrition and health in developing countries. Can Med Association Journal173. 2005:279–86. 10.1503/cmaj.050342 PMC1180662 16076825

[6] Younis K, Ahmad S, Badpa A (2015). Malnutrition: causes and strategies. J Food Process Technol.

[7] El-Ghannam AR (2003). The global problems of child malnutrition and mortality in different world regions. J health social policy.

[8] Houweling TA, Kunst AE, Looman CW, Mackenbach JP (2005). Determinants of under-5 mortality among the poor and the rich: a cross-national analysis of 43 developing countries. Int J Epidemiol.

[9] Firaol L, Rahel M, Gebi H, Tewodros D et al. Under-five mortality and associated factors in southeastern Ethiopia,Plos one, 2021. 10.1371/journal.pone.0257045 PMC8423275 34492085

[10] Belete A, Sofonyas A, Melkalem M et al. Determinants of under-five mortality in Ethiopia using the recent 2019 Ethiopian demographic and health survey data: nested shared frailty survival analysis, Archives of Public Health, 137 (2022). 10.1186/s13690-022-00896-1 PMC9099053 35562788

[11] Antai D (2011). Regional inequalities in under-5 mortality in Nigeria: a population-based analysis of individual-and community-level determinants. Popul health metrics.

[12] Nure Alam S, Nuruzzaman H, Abdul G (2011). Differentials and determinants of under-five mortality in Bangladesh. Int J Curr Res.

[13] Mahfouz MS, Surur AA, Ajak DAA, Eldawi EA (2009). Level and determinants of infant and child mortality in Malakal Town-southern Sudan. Sudan J public health.

[14] Ettarh R, Kimani J (2012). Determinants of under-five mortality in rural and urban Kenya. Rural Remote Health.

[15] Xu Y-H, Huang X-W, Yang R-L (2011). The under-five mortality rate and the causes of death in Zhejiang Province between 2000 and 2009. Zhongguo dang dai er ke za zhi = Chinese. J Contemp Pediatr.

[16] Ayele DG, Zewotir TT, Mwambi H (2017). Survival analysis of under-five mortality using Cox and frailty models in Ethiopia. J Health Popul Nutr.

[17] Yaya S, Bishwajit G, Okonofua F, Uthman OA, van Wouwe JP. “Under five mortality patterns and associated maternal risk factors in sub-Saharan Africa: A multi-country analysis,” PLoS ONE, vol. 13, no. 10, article no. e0205977, 2018. 10.1371/journal.pone.0205977 PMC6201907 30359408

[18] Ababa A, Calverton E. Central statistical agency (Ethiopia) and ICF international. Ethiopia and Calverton: Ethiopia Demographic and Health Survey. 2011.

[19] Tefera YG, Ayele AA (2021). Newborns and Under-5 mortality in Ethiopia: the necessity to revitalize Partnership in Post-COVID-19 Era to meet the SDG targets. J Prim Care Community Health.

[20] Getachew Y, Bekele S (2016). Survival analysis of under-five mortality of children and its associated risk factors in ethiopia. J Biosens Bioelectron.

[21] Geremew BM, Gelaye KA, Melesse AW, Akalu TY, Baraki AG. Factors Affecting Under-Five Mortality in Ethiopia: A Multilevel Negative Binomial Model.Journal of pediatric-health-medicine-and-therapeutics 2020:11. 10.2147/PHMT.S290715 PMC7781031 33408551

[22] Nasejje JB, Mwambi HG, Achia TN (2015). Understanding the determinants of under-five child mortality in Uganda including the estimation of unobserved household and community effects using both frequentist and bayesian survival analysis approaches. BMC Public Health.

[23] Kazembe L, Clarke A, Kandala N-B. Childhood mortality in sub-Saharan Africa: cross-sectional insight into small-scale geographical inequalities from Census data. BMJ Open 2012;2(5) 10.1136/bmjopen-2012-001421 PMC3488715 23089207

[24] Adedini SA (2015). Unmet need for family planning: implication for under-five mortality in Nigeria. J Health Popul Nutr.

[25] Gizachew G (2021). Determinant factors of under-five mortality in Southern Nations, Nationalities and People’s region (SNNPR), Ethiopia. Ital J Pediatr.

[26] Machado CJ, Hill K (2003). Determinants of neonatal and post-neonatal mortality in the City of São Paulo. Rev Bras Epidemiol.

[27] Omariba DWR, Beaujot R, Rajulton F. Determinants of infant and child mortality in Kenya: an analysis controlling for frailty effects. Popul Res Policy Rev 2007.

[28] Orji EO, Ogunniyi SO, Onwudiegwu U. Beliefs and perceptions of pregnant women at Ilesa about caesarean section. Trop J Obstet Gynaecol 2003;20.

